# Nitrite Biosensing via Selective Enzymes—A Long but Promising Route

**DOI:** 10.3390/s101211530

**Published:** 2010-12-15

**Authors:** M. Gabriela Almeida, Alexandra Serra, Celia M. Silveira, Jose J.G. Moura

**Affiliations:** 1 REQUIMTE—Departmento de Química, Faculdade de Ciencias e Tecnologia (UNL), 2829-516 Monte Caparica, Portugal; E-Mails: a.serra@dq.fct.unl.pt (A.S.); celia.silveira@dq.fct.unl.pt (C.M.S.); jose.moura@dq.fct.unl.pt (J.J.G.M.); 2 Escola Superior de Saude Egas Moniz, Campus Universitario, Quinta da Granja, 2829-511 Monte Caparica, Portugal

**Keywords:** nitrite, biosensors, nitrite reductases, electrochemical transducers

## Abstract

The last decades have witnessed a steady increase of the social and political awareness for the need of monitoring and controlling environmental and industrial processes. In the case of nitrite ion, due to its potential toxicity for human health, the European Union has recently implemented a number of rules to restrict its level in drinking waters and food products. Although several analytical protocols have been proposed for nitrite quantification, none of them enable a reliable and quick analysis of complex samples. An alternative approach relies on the construction of biosensing devices using stable enzymes, with both high activity and specificity for nitrite. In this paper we review the current state-of-the-art in the field of electrochemical and optical biosensors using nitrite reducing enzymes as biorecognition elements and discuss the opportunities and challenges in this emerging market.

## 1. Introduction

Fast-growing biosensor technology has broad applications in the fields of health care, agricultural, environmental and industrial monitoring and nitrite biosensors are no exception. Clear-cut markets for nitrite sensing exist in the food industry, pollution control and clinical diagnostics. This review aims to provide a global overview of the efforts done towards the development of efficient nitrite biosensors using redox enzymes with catalytic activity for this analyte. Although attention was mainly focused on nitrite reductases, the article also covers parallel studies conducted with other proteins that display a secondary catalytic activity for nitrogen oxides. Due to the electron transfer (ET) nature of the catalysed reactions, signal transduction was achieved through electrochemical approaches with only few exceptions.

The article begins by highlighting the relevance of nitrite quantification in real samples, proceeds with a detailed description of relevant works on nitrite biosensing and ends with a brief insight on future trends.

### 1.1. Addressing the Problem of Nitrite Assessment

Nitrates (NO_3_^−^) and nitrites (NO_2_^−^) are frequently present in plants, soils and waters; since their chemistries are practically indissociable, one is rarely found without the other. If in excessive levels, these ions can have an adverse impact on public health and ecological systems. Nitrite is the foremost toxic agent, but the fairly inert nitrate is easily reduced to nitrite by bacterial action in the soil or within the digestive system [1,2]. In recent years, in order to manage environmental and health risks caused by nitrates/nitrites exposure, the most important governmental agencies have promulgated rules and directives to restrict the level of these ions in drinking waters and food products. Furthermore, the determination of nitrites in human physiological fluids is commonly used for clinical diagnosis [3].

#### Environmental Risks

The anthropogenic input of nitrites and nitrates to the environment can occur through the photochemical conversion of atmospheric nitrogen oxides (NO_x_) generated by all sorts of combustion processes (industrial, domestic and automobile) [1,4,5]. Applications of nitrites in textile, metal, petroleum and pharmaceutical industries are also documented [6,7]. As for nitrates, the most important pollution source arises from the intensive use of *N*-fertilizers in farming activities; though, anaerobic conditions may favor their conversion into nitrites [2]. Besides unbalancing the biogeochemical nitrogen cycle by interfering with the denitrification/nitrification processes [4,8] the most serious ecological repercussion of excessive nitrates/nitrites levels comes from the contamination of groundwater supplies by percolation of these highly soluble ions through natural aquifers. In conjunction with phosphorous fertilizers, the nitrate enrichment of surface waters is also responsible for the eutrophication of aquatic ecosystems and contamination of fish and shellfish cultures [2,8,9]. According to the latest European Commission’s report [COM(2010)47] on the implementation of directive 91/676/EEC (aimed to protect surface and ground waters against nitrate originated from agricultural fertilizers) nitrate pollution has been falling. Nevertheless, further efforts are still required to meet water quality standards across the European Union.

#### Health Hazards

Nitrites are found in vegetables only in very small quantities whilst nitrates occur naturally in most eaten vegetables in highly variable amounts; lettuce, spinach, beetroot, chervil, cress and celery are nitrate-rich vegetables. Yet, high contents of nitrates are typically observed if plants are grown in soils overloaded with nitrogen fertilisers [10–13]. Along with nitrate salts (E251, E252), nitrite potassium and sodium salts (E249, E250) have been largely used in the manufacturing of processed and cured meats, fishes and some cheeses, with the double role of protecting consumers from pathogenic microorganisms such as *Clostridium botulinum* and enhancing organoleptic properties as colour and flavour [14,15]. Human exposure to NO_3_^−^/NO_2_^−^ ions results largely from dietary ingestion of these food products. Only a minor percentage comes from drinking water where the levels of these compounds usually comply with regulation (see below) [2,10].

Methemoglobinemia is the principal adverse health effect caused by excessive nitrates/nitrites intake. Nitrite can irreversibly oxidize hemoglobin to methemoglobin which is unable to bind oxygen, causing clinical cyanosis among other symptoms. Infants are particularly susceptible to nitrite induced methemoglobinemia, often referred to as the *blue-baby* syndrome; a small number of fatal cases has been reported, generally associated to the consumption of water resources that failed drinking water standards [2,10]. Concern has been raised on the potential role of nitrite in forming carcinogenic *N*-nitroso compounds (NOCs) via reaction with secondary amines [16,17]. Although many NOCs have been shown to be genotoxic in animal models, the relationship between nitrites/nitrates intake and the risk of cancer in humans has not been unequivocally proved so far [10]. No matter the controversy, the information generated from the analytical surveillance of nitrite in food products is fundamental for the management of health risks.

The issue of nitrates/nitrites toxicity led to the implementation of rules to restrict their level in drinking waters and foodstuffs. European directive 98/83/EC has established the maximum admissible levels of nitrate and nitrite in drinking water at 50 and 0.1 ppm, respectively. Likewise, the World Health Organization (WHO/SDE/WSH/07.01/16) has set these limits at 50 ppm (NO_3_^−^) and 3 ppm (NO_2_^−^). More recently, following the European Food Safety Authority recommendations, 2006/52/EC directive has reduced the authorized levels for nitrites and nitrates in meat and other food products, which should be controlled on the basis of *added* rather than *residual* amounts (e.g., 150 mg/kg of nitrites in meat products).

#### Physiological Aspects

Nitrates and nitrites are also present in mammalian physiological systems, either from dietary provision or endogenous formation. The chemical reactions of these ions in the physiological environment are strongly related to the nitric oxide (NO) metabolism; the relationship between the three NO_x_ species is a current matter of intense research and was recently reviewed by Hord *et al.* [10] and Lundberg *et al.* [18]. Nitric oxide is a crucial mediator in cell signal transduction that plays a critical role in numerous physiological processes and in the pathophysiology of many human diseases. Indeed, NO has a major role in regulating cardiovascular functions and modulating inflammatory, infectious and degenerative disorders. As a consequence, abnormal production of NO has been implicated in a number of pathological conditions, such as acute lung disease, atherosclerosis and septic shock [19,20]. Nitric oxide has a short lifetime of only a few seconds, so endogenous NO formation is indirectly assessed by quantifying its stable metabolites, nitrate and nitrite [3,20,21]. However, nitrate concentration in plasma does not show great variations over acute nitrosative stress and is rather affected by exogenous intake and renal function [20]. Therefore, plasma and urine nitrite measurements are usually carried out for diagnosing and/or monitoring patients with conditions as infection, rejection and inflammation [3]. Values reported in the literature for basal nitrite plasma are quite divergent, probably due to variations in blood sampling, sample processing and limitations of the analytical methods which do not allow the accurate measurement of nitrites in complex matrices. For this reason, Dejam *et al.* have designed an experimental methodology to prevent nitrite oxidation/reduction in collected samples using a ferrycianide-based hemoglobin oxidation solution. Accordingly, plasma nitrite concentration is in the nanomolar range (*ca*. 100 nM) [22].

In recent years, nitrite itself was also recognized as having its own intracellular signaling role in mammalian physiology [20,23] and the concept of a potential therapeutic use of nitrates/nitrites in human health has emerged afterwards [10,18]. These current trends lead us to conclude that monitoring of these anions in physiological systems is also relevant from a purely academic standpoint.

### 1.2. Enzyme Based Biosensors as Key Enablers of Accurate Nitrite Determination

At this point, it is worth noting that the total concentration of NO_3_^−^/NO_2_^−^ is usually assessed by previous reduction of nitrates into nitrites either by enzymatic (e.g., nitrate reductase) or inorganic (e.g., copper/cadmium columns) processes. This strategy is also followed to determine individual nitrates contents by subtracting the total amount of NO_3_^−^/NO_2_^−^ from the nitrites concentration determined in non reduced samples [2,21].

The numerous analytical assays proposed for nitrite quantification encompass sophisticated and centralized techniques such as UV-Vis spectrophotometry (Griess reaction), ion chromatography, polarography, capillary electrophoresis, gas chromatography coupled to mass spectrometry (GC-MS) or fluorescence spectrophotometry. However, most of these analytical methods have shown important limitations such as sample pre-treatment, susceptibility to matrix interferences, insufficient detection limits, long time of analysis and lack of portability (the interested reader may consult the comprehensive reviews [1–3,21,24–29] and references cited therein). This situation has challenged Analytical Sciences to develop innovative and improved tools, leading to the budding field of enzyme based nitrite biosensors. Consequently, major progresses have been made in nitrites analysis over the last fifteen years, through the employment of highly selective, active and stable nitrite reducing enzymes isolated from bacterial sources. Besides giving the required selective and sensitive response, biosensors should be easy and cheap to fabricate in miniature dimensions, turning a long and elaborated laboratory protocol into a simple task, quickly executed onsite. Furthermore, as reagentless devices, nitrite biosensors may be considered environmentally friendly analytical tools.

After an extensive literature survey, we have identified a large variety of protein electrodes which are able to perform nitrite quantification. In line with the selectivity of the biorecognition event, we have decided to separate such nitrite biosensors in two different groups hereafter designated as *non specific proteins* and *nitrite reductases*.

As transducing modes one could find a vast predominance of electrochemical approaches, the largest group being the voltammetric and/or amperometric ones and a small number being based on potentiometric or conductimetric platforms. Alternatively, the spectroscopic changes that take place during the catalytic cycle were also employed in the construction of optical biosensors. The strategies proposed for protein immobilization have relied on a variety of materials, ranging from non-conducting polymers, electropolymerized films, redox active clays, sol-gel silica glasses, carbon nanotubes, metal nanoparticles and DNA tethers, either alone or in composite formulations.

**Figure 1.**
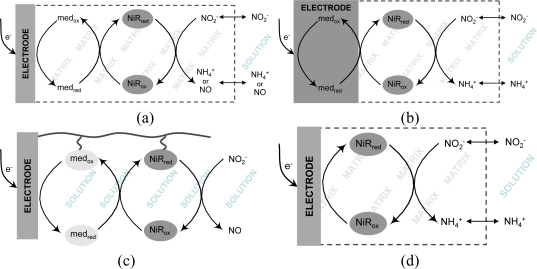

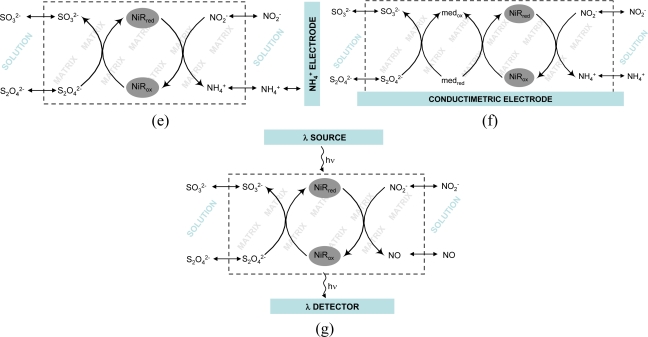
Schematic representations of the working principles of different enzymatic nitrite biosensors. Mediated amperometric transduction: **(a)** mediator and enzyme are co-immobilized on the matrix film; **(b)** mediator is entrapped on the working electrode material; **(c)** enzyme and physiological mediator linked to a DNA modified electrode. **(d)** Direct electrochemical transduction; **(e)** Potentiometric transduction; **(f)** Conductimetric transduction; **(g)** Optical transduction (med_ox_—mediator in the oxidized form; med_red_—mediator in the reduced form; NiR_ox_—NiR oxidized state; NiR_red_—NiR reduced state; dithionite (S_2_O_4_^2−^) works as reducing equivalents source).

**Figure 2.**
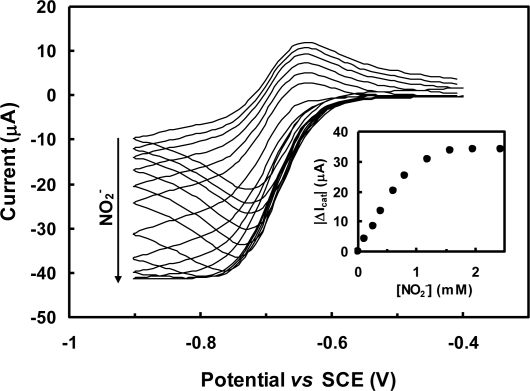
Cyclic voltammograms recorded with a Nafion/c*c*NiR/methyl viologen/GCE biosensor in the absence of nitrite and in the presence of various nitrite concentrations; adapted from [30]. Inset: corresponding calibration curve.

The implementation of a biosensing system is not simply a matter of finding a suitable enzyme and attaching it onto a transducing surface. It usually faces several obstacles that start from the firm immobilization of the protein without denaturation or leakage. Very often, the bottleneck of electrochemical biosensors is the achievement of an efficient electronic communication between proteins and electrodes. In the context of nitrite biosensors, the majority of proposals have employed redox mediators (e.g., viologen derivatives) which display a fast and reversible electrochemical response and are able to shuttle electrons rapidly to the protein redox centers, following the transducing schemes depicted in Figures 1(a–c). In accordance to an EC’ mechanism, the increase in cathodic peak current resulting from the regeneration of the oxidized mediator by the catalytic reaction step is directly related to the amount of substrate being processed, as exemplified in the cyclic voltammograms shown in Figure 2. Very recently, mediatorless approaches based on the direct electron transfer (DET) between the redox active enzyme and the electrode material were also reported (Figure 1(d)). In this situation, the increase in catalytic currents results from the direct regeneration of enzyme cofactors.

Besides dictating the efficiency of electrochemical transduction, the design of biosensors has also played a fundamental role in defining the response features. For example, sensitivity and response time, which strongly depend on mass transfer limitations, were particularly influenced by the characteristics of the immobilization matrix.

The next section aims to provide a description of most works reported so far, with special emphasis on devices based on *nitrite reductases*. This major group was sub-divided according to the degree of integration and communication with the electrode. Attention should be given to the fact that the direct comparison of the analytical properties associated to each proposal is not straightforward since the assessment of relevant parameters such as stability and selectivity lacked standardization. Moreover, if a full comparison is required, experimental conditions that may vary from system to system (e.g., temperature, pH or ionic strength) should also be taken into account.

## 2. Amperometric and/or Voltammetric Biosensors

### 2.1. Proteins with non Specific Activity for Nitrite

Myoglobin (Mb) and hemoglobin (Hb) are oxygen binding proteins. Hb is found in the erythrocytes of numerous vertebrates and invertebrates where it combines reversibly with O_2_ and transports it to the cells [31,32]. Mb exists mainly in muscle tissue where it serves as an intracellular storage site for oxygen [33].

Horse heart Mb and bovine blood Hb have been commonly used as model proteins for testing new immobilization materials with prospective applications in the construction of electrochemical biosensors. Most of the works reported in the literature aimed the detection of hydrogen peroxide (H_2_O_2_) through a direct electrochemical response. The ability of such bioelectrodes to also detect nitrite ions was considered by most authors as a secondary catalytic activity, quite often insufficiently described [34–50]. In the same context, but in a less extent, catalase (Cat) from bovine liver and horseradish peroxidase (HRP) have also revealed catalytic activity for nitrite [46,51].

It is frequently proposed that it is not nitrite but nitric oxide (NO) generated by nitrite disproportionation (Equation 1) that directly interacts with the active site of these proteins [52,53]:

(1) $$3{\text{NO}}_{2}^{-}+{\text{H}}_{2}\text{O}↔2\text{NO}+{\text{NO}}_{3}^{-}+2{\text{OH}}^{-}$$

In fact, the great majority of heme proteins is well known for its capacity to easily bind and eventually react with NO [54]. Therefore, the shape of the cyclic voltammograms displayed by the immobilized proteins, either or not in the presence of nitrite, can be explained by the currently accepted reaction mechanism for the bioelectrocatalytic reduction of NO, which can be described by the following sequence of chemical equations:

(2) $$\text{heme}-\text{Fe}(\text{III})+{\text{e}}^{-}\to \text{heme}-\text{Fe}(\text{II})$$

(3) heme-Fe(II)+NO→heme-Fe(II)−NO

(4) $$2\;\text{heme}-\text{Fe}(\text{II})-\text{NO}+2{\text{e}}^{-}+2{\text{H}}^{+}\to 2\;\text{heme}-\text{Fe}(\text{II})+{\text{H}}_{2}\text{O}+{\text{N}}_{2}\text{O}$$

A reversible system of two peaks located in the range 0.08–0.12 V *vs.* NHE is assigned to the heme-Fe(III)/Fe(II) redox couple (Equation 2). When nitrous oxide binds to the ferrous heme (Equation 3), a second irreversible reduction peak is observed between −0.45 V and −0.72 V *vs.* NHE (Equation 4) featuring a catalytic behavior [34–36,38,40,46]. Oxygen purging from solutions is therefore, mandatory. Interestingly, however, Zhao *et al.* [37] and Sun *et al.* [39] have detected the development of catalytic currents associated to the Mb-Fe(III) reduction peak (Equation 2), whereas Liu *et al.* [41] mentioned a similar behavior for Hb at low nitrite concentrations. Yet, none of these authors could find an explanation for the fact. Alternative reactions routes for the electrocatalytic reduction of NO_x_ compounds by heme models and proteins have been also proposed and were broadly reviewed by Blair and co-workers [55].

Amperometric biosensors for NO_2_^−^ determination using cytochrome *c* (cyt-*c*) from horse heart as biological component have also been recently reported [56,57]. These sensors explore the catalytic oxidation of nitrite to nitrate through a highly reactive form of the protein (so-called π-cation), according to the following reaction scheme:

(5) $$\text{cyt}-c\;\text{Fe}(\text{II})\to \text{cyt}-c\;\text{Fe}(\text{III})+{\text{e}}^{-}$$

(6) $$\text{cyt}-c\;\text{Fe}(\text{III})\to {\left[\text{cyt}-c\;\text{Fe}{\left(\text{IV}\right)}^{+}\right]}^{•}+2{\text{e}}^{-}$$

(7) $${\left[\text{cyt}-c\;\text{Fe}{\left(\text{IV}\right)}^{+}\right]}^{•}+{\text{NO}}_{2}^{-}+{\text{H}}_{2}\text{O}\to \text{cyt}-c\;\text{Fe}(\text{III})+{\text{NO}}_{3}^{-}+2{\text{H}}^{+}$$

The main drawback of this strategy is the high working potential required for generating the reactive radical species (*ca.* 0.6 V *vs.* SCE) and monitoring the catalytic reaction (>0.7 V *vs.* SCE), eventually allowing unspecific oxidation reactions in real samples [56,57].

Table 1 summarizes some of the most recent and representative works done in the field. A detailed analysis of this table clearly demonstrates that although electrode modifications are made with all sorts of materials, combined with polymers, silicates, surfactants or ionic liquids, the systems share many common features. Direct electron transfer between proteins and electrodes is frequently promoted by the modifying matrix and the intensity of the catalytic currents is correlated with nitrite concentration; amperometric transduction is a frequent option; and the working electrodes are always made of carbon materials (usually, glassy carbon (GC)). A different concept was recently proposed by Chen *et al.* [58] who have designed a complex transducing scheme that sequentially associates the two hydrogen peroxide (H_2_O_2_) dependent enzymes HRP and Cat. In the presence of nitrite, the H_2_O_2_ decomposition by the catalase activity is inhibited, thus increasing the amount of H_2_O_2_ available for reduction at the HRP wired electrode [58].

Overall, the tabulated analytical parameters were highly variable. For instance, the detection limit can be as low as 0.06 μM and go up to 700 μM. Though, the substrate promiscuity of all these heme containing proteins puts in risk the selectivity of determination, so they are not recommended for the construction of a selective nitrite biosensor. Instead, much more selective biocatalysts should be employed. The next section aims to describe all nitrite biosensing systems reported hitherto that make use of highly selective enzymes for nitrite reduction.

### 2.2. Nitrite Reductases

**Figure 3.**
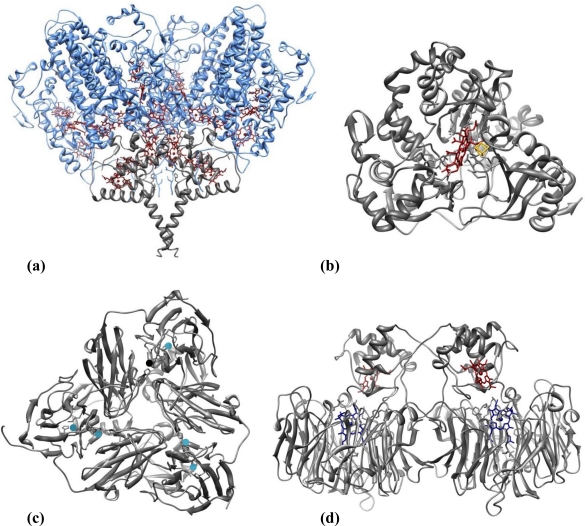
Three-dimensional structures of nitrite reductases. **(a)** *Desulfovibrio vulgaris* Hildenborough multiheme *c* nitrite reductase (NrfA_4_NrfH_2_ complex); the catalytic subunit (NrfA) is depicted in blue and the electron donor subunit (NrfH) in gray; heme groups are shown in dark red [65]. **(b)** Spinach nitrite reductase; siroheme is shown in dark red and iron-sulfur cluster in yellow [70]. **(c)** *Achromobacter cycloclastes* copper nitrite reductase (trimer); the copper centres are shown in blue [71]. **(d)** *Pseudomonas aeruginosa* cytochrome *cd*_1_ nitrite reductase (dimer); heme *c* is depicted in dark red and heme *d* in blue [72].

Nitrite reducing enzymes (NiRs) are innate candidates for playing the role of biorecognition elements in nitrite biosensing devices. Figure 3 illustrates representative three-dimensional structures of the four classes of NiRs so far recognized, which are grouped according to the type of co-factors or reaction product (see below). The topic of nitrite reductases enzymes (structure, reactivity and biological function) has been reviewed by several authors as Richardson *et al.* [59], Einsle *et al.* [60] and Moura *et al.* [61], to cite a few. Examples of the four types of NiRs were already featured in biosensor applications.

#### Ammonia Forming Nitrite Reductases

Two different NiRs are able to catalyse the six electron reduction of nitrite to ammonia (NH_4_^+^), according to the following equation:

(8) $${\text{NO}}_{2}^{-}+8{\text{H}}^{+}+6{\text{e}}^{-}\to {\text{NH}}_{4}^{+}+2{\text{H}}_{2}\text{O}$$

The dissimilatory cytochrome *c* nitrite reductases (c*c*NiR) isolated from sulfate or sulfur reducing bacteria are multi-heme enzymes involved in a respiratory process that represents an important branch of the biological nitrogen cycle. c*c*NiRs can also use nitric oxide (NO) and hydroxylamine (NH_2_OH) as substrates [62,63]. They are usually isolated from the periplasmic membrane as high molecular mass multimers, comprising the subunits NrfA (61 kDa) and NrfH (19 kDa) [64] in a NrfA_4_NrfH_2_ stoichiometry (Figure 3(a)) [65]. The small hydrophobic tetrahemic polypeptide NrfH is the physiological electron donor of NrfA. The latter houses five hemes per monomer in a tight packing, so rapid ET rates can be achieved. All but the active centre are hexa-coordinated *c*-type hemes whereas the catalytic heme is bound to a lysine as the fifth coordinate ligand and has the sixth axial position available for substrate binding [64–67].

The other sort of ammonia forming NiRs belong to the ferredoxin-dependent nitrite reductases family, commonly isolated from photosynthetic organisms such as plants, algae and cyanobacteria. These assimilative enzymes are constituted by a single polypeptide chain (60–65 kDa) composed of three domains folded around the two prosthetic groups—a siroheme and an iron-sulfur cluster [4Fe-4S]—which are connected by a bridging sulfur cysteine. The siroheme group is the nitrite binding site (Figure 3(b)) [68–70].

#### Nitric Oxide Forming Nitrite Reductases

Two types of dissimilatory NiRs involved in bacterial denitrification (one of the main branches of the global nitrogen cycle) catalyse the one-electron reduction of nitrite to nitric oxide (Equation 9). They are known as copper-containing nitrite reductases (CuNiRs) and cytochrome *cd*_1_ nitrite reductases (*cd*_1_NiRs).

(9) $${\text{NO}}_{2}^{-}+2{\text{H}}^{+}+{\text{e}}^{-}\to \text{NO}+{\text{H}}_{2}\text{O}$$

CuNiRs (Figure 3(c)) are trimer proteins composed of three identical subunits (3 × 37 kDa), with two coper ions each; type-I copper centre is involved in electron transfer, while type-II copper centre constitutes the catalytic centre [71,73,74]. *cd*_1_NiRs are soluble, homodimeric (2 × 60 kDa) proteins; each subunit contains a *c*-type and a *d*_1_ heme, which constitute the electron acceptor and the substrate-binding sites, respectively (Figure 3(d)) [75,76]. This enzyme can also catalyse the two-electron reduction of hydroxylamine to ammonia [77] and the four-electron reduction of oxygen to water [78] *in vitro*.

#### 2.2.1. Biosensors Based on Mediated Electrochemistry

##### 2.2.1.1. Synthetic Mediators

Several studies have been described in the literature concerning the mediated electrochemistry of different NiRs. In this context, a wide range of synthetic electron carriers, either in solution or immobilized, were tested prospecting the development of mediated nitrite biosensing devices [79–81]. Although NiRs are able to accept electrons from a variety of redox mediators, electron donors with quite negative redox potentials such as viologen dyes, are usually chosen since the biosensor analytical performances are superior [30,79,82,83]. Yet, the high polarization potentials required complicate biosensor operation, given that side reactions (inevitably from oxygen, for example) will interfere in the analytical process. For this reason, nitrite biosensors based on reducing enzymes are almost exclusively anaerobic devices, *i.e.*, they should be employed in previously degassed solutions. Whenever possible it is advisable to select mediators with higher potentials. Phenazines [80,81,84], safranines [80] and anthraquinones [85] are such examples featured in NiR based biosensors.

###### Non-Integrated Systems:

A biosensor is generally described as a contained unit which integrates a biorecognition element in direct physical contact with a signal transducer element [86,87]. In a mediated biosensor the electron carrier should also make part of the biorecognition system. For this reason, it must be immobilized in conjunction with the biological component. Hence, we have not considered nitrite biosensing proposals in which the electron donor is added to the electrolyte solution as real biosensors. Such *non-integrated systems* are, nonetheless, worth mentioning efforts that somewhat inspired further studies on the development of electrochemical enzyme devices for nitrite quantification.

Strelihtz and co-workers described this type of system with the *cd*_1_NiR from *Paracoccus denitrificans* (*P. denitrificans*) in two separate occasions [80,81]. The enzyme was physically entrapped on graphite electrodes with dialysis membranes, while amperometric measurements were done using mediating species in solution. A broad list of synthetic electron donors was tested to determine the most appropriate ones to be used on electrochemical biosensor devices. The highest currents for nitrite reduction were obtained with phenazines, methyl viologen and bromophenol dyes. Since 1-methoxy-5-methylphenazinium methylsulfate (1-methoxy PMS) has the highest reduction potential, it was chosen for the further calibrations [80,81]. Sasaki *et al.* followed a similar approach using a recombinant CuNiR from *Alcaligenes faecalis* S-6 (*A. faecalis*) overexpressed in *Escherichia coli* (*E. coli*), which has the double of the enzyme activity displayed by the wild type protein [84]. The enzyme was entrapped on a gold surface with filter paper and a dialysis membrane. The mediator 1-methoxy PMS was added to the supporting electrolyte at a fixed concentration. Amperometric measurements with varying concentrations of nitrite were made at −150 mV (*vs.* Ag/AgCl) following the 1-methoxy PMS reduction. Some problems were encountered on the sensor tests with rain waters, most likely as a consequence of an insufficient solution deoxygenating. The stability of the electrode was rather poor, probably due to the weak immobilization method [84]. The reaction taking place in the systems described above is represented by Equation 10:

(10) $${\text{NO}}_{2}^{-}+2{\text{H}}^{+}+1-\text{methoxy}\;\text{PMS}\to \text{NO}+{\text{H}}_{2}\text{O}+1-\text{methoxy}\;{\text{PMS}}^{+}$$

In another work, the c*c*NiR from *Desulfovibrio desulfuricans* ATCC 27774 (*D. desulfuricans*) was entrapped within a polyacrilamide gel which was polymerized atop a GC electrode [88]. Voltammetric nitrite dependent catalytic currents were observed through methyl viologen (MV) mediation, which was present in the electrolyte solution (Equation 11). A linear current response could be obtained up to 200 μM. This electrode kept a stable response for at least one day. It was used for nitrite quantification in real samples of bacterial liquid growth media. The results were in good agreement with the control method (HPLC) [88]. Table 2 compiles the analytical characteristics of these non-integrated systems, among others.

(11) $${\text{NO}}_{2}^{-}+8{\text{H}}^{+}+6{\text{MV}}^{+}\to {\text{NH}}_{4}^{+}+2{\text{H}}_{2}\text{O}+6{\text{MV}}^{2+}$$

###### Integrated Systems

The co-immobilization of enzymes and mediators is a key factor to take into account when designing a biosensor. From the proteins standpoint, it is important to protect them from harsh environments which can cause losses of activity or denaturation. Regarding mediators, these usually small molecules can easily diffuse through membranes, and thus it is necessary to find strategies to retain them conveniently on the sensor, avoiding instability setbacks.

Simultaneous entrapment of enzymes and mediators in polymeric matrices is a common methodology used in biosensors and nitrite biosensors are no exception. In fact, several proposals based on casting or electropolymerization of polymer/enzyme mixtures on electrode surfaces have been reported, as described below.

A straightforward method for the preparation of enzyme modified electrodes consists of casting a mixture of the protein and a *non-conducting polymer* on the electrode surface and allowing it to polymerize or simply dry. Such strategy was followed in a study published by Strehlitz *et al.* [80] where a poly(carbamoyl sulfonate) hydrogel mixed with c*c*NiR from *Sulfurospirillum deleyianum* (*S. deleyianum*), was deposited over the transducing electrode. The electron mediator, phenosafranin, was integrated on carbon paste working electrodes; in this way, a true mediated biosensor configuration was created for the first time, since the electron carrier was incorporated on the transducer. Figure 1(b), represents the working principle of this biosensor, whereas Equation 12 describes the corresponding chemical reactions. Amperometric measurements were made at −600 mV (*vs.* SCE) and the corresponding calibration data is listed in Table 2. The biosensor’s response time was quite long, taking 3 minutes to reach a 95% value, fact that was attributed to the thickness of the hydrogel membrane. The response decreased about 50% if measurements were made in air-saturated solutions clearly indicating major oxygen interferences. As so, solutions had to be degassed prior to analysis. Stability studies showed that the c*c*NiR retained about 85% of activity after 8 days of storage. Nonetheless, if the biosensor was frequently used, enzyme deactivation occurred and the biosensor lost 50% of activity after only 17 hours [80]:

(12) $${\text{NO}}_{2}^{-}+8{\text{H}}^{+}+3{\text{PSH}}_{2}^{+}\to {\text{NH}}_{4}^{+}+2{\text{H}}_{2}\text{O}+3{\text{PS}}^{+}+6{\text{H}}^{+}$$

More complex casting designs were described by Quan *et al.* for CuNiR (*Rhodopseudomonas sphaeroides* (*R. sphaeroides*)) based nitrite biosensors [79], and by our group for c*c*NiR (*D. desulfuricans*) containing devices [30]. The same redox mediator (methyl viologen) was employed in both cases and its immobilization was based on electrostatic interactions with the polymeric layers. Both sensors operate according to the reaction scheme illustrated in Figure 1(a). In the CuNiR biosensing system, after casting on top of a GC surface a mix solution composed of redox mediator, enzyme and polymer (poly(vinyl alcohol)), a second layer of poly(allylamine hydrochloride) was applied, which helped preventing mediator leakage (this positively charged film could maintain the cationic electron donor on the sensor by electrostatic repulsion). The biosensor preparation was completed by the addition of a hydrophilic polyurethane coat. Equation 13 represents the chemical reaction taking place in the presence of nitrite. Amperometric measurements were done at −750 mV (*vs.* Ag/AgCl), thus requiring solution degassing. A response time (*t*_90%_) of 20 s was obtained with this sensor. The reproducibility was low (8.2%) probably due to the high number of components present; nevertheless, variability within the same set of sensors was around 3.8%. The biosensor was prone to a strong interference from nitrate (38% of the sensitivity obtained for nitrite). Storage stability, determined as maintenance of 80% of the sensors initial activity, was 24 days [79].

(13) $${\text{NO}}_{2}^{-}+2{\text{H}}^{+}+{\text{MV}}^{+.}\to \text{NO}+{\text{H}}_{2}\text{O}+{\text{MV}}^{2+}$$

In our own work, a composite Nafion/c*c*NiR film was firstly formed on top of a GC electrode and then immersed on a mediator containing solution, allowing it to incorporate the ionomeric polymer. The cationic exchange nature of Nafion was in this way exploited to accumulate methyl viologen on the electrode surface. The biosensor was characterized by cyclic voltammetry, in oxygen free solutions. The stability of this configuration was somewhat compromised given that there was a progressive drop in the mediator levels due to its leakage to the bulk solution. A reproducible response could nonetheless be obtained for two days, if matrix mediator levels were kept constant by re-immersing the electrode in methyl viologen solution. Biosensor activity was short lived, completely ceasing after eight days. Yet, this low stability was most likely related with the enzyme’s progressive loss of activity rather than the mediator quantity on the sensor. Additional diffusion constrains imposed by Nafion’s anionic nature probably contributed for the wide linear range (Table 2) [30].

Enzyme incorporation into *redox active matrices* is another methodology used in the construction of electrochemical biosensors. The enzymes are entrapped and at the same time electrically wired, so a better electrical connection is achieved. As a consequence, resulting biosensors are usually very sensitive.

A redox active [ZnCr-AQS] layered double hydroxide (LDH), an anionic exchange material doped with anthraquinone-2-monosulfonate (AQS), and an electropolymerizable poly(pyrrole-viologen) (PPyV) have been successfully employed on the encapsulation of *D. desulfuricans* c*c*NiR [82,85]. In the first system, GC electrodes were modified with mixtures of enzyme and LDH and their response to nitrite was evaluated by amperometric titration at −600 mV (*vs.* Ag/AgCl) following the AQS reduction (see Equation 14 and Figure 1(a)). Highly sensitive nitrite detection was achieved, within a very short response time (<5 s). The detection limit was the lowest ever presented for this type of device, reaching 4 nM; the sensitivity (1.8 A M^−1^ cm^−2^) of the calibration plot (linear from 0.015–2.35 μM) was also one of the highest reported values (see Table 2). The LDH was considered as a promising biocompatible matrix, since the electrode kept up to 60% of its initial response to nitrite after 32 days [85]. In a second study, a mixture of c*c*NiR and water dispersed monomers of *N*-methyl-*N*’-(12-pyrrol-1-yldodecyl)-4,4′-bipyridinium ditetrafluoroborate was deposited on GC surfaces and subsequently electro-polymerized by controlled-potential electrolysis. In this way, the viologen groups (V) were covalently attached to the polypyrrole backbone (PPyV), so the polymer played the double role of protein immobilization and redox mediation via an electron hopping mechanism, *i.e.*, viologens groups are reduced at the electrode surface and shuttle electrons to c*c*NiR, which in turn reduces nitrite to ammonia (Figure 1(a) and Equation 15) [82]. Wu *et al.* [83] have used a similar strategy with the CuNiR of *R. sphaeroides* which was immobilized on a GC electrode modified with a pyrrol electropolymerized film of *N*-(3-pyrrol-1-yl-propyl)-4,4′-bipyridinium (PPB) and poly(benzyl viologen) (PBV). The PPB layer in this sensor also worked as binding matrix and electron mediating system via the pyridinium groups (Figure 1(a) and Equation 16). The NiR utilized in this configuration was modified with a maltose-binding protein domain and overexpressed in *E. coli.* This modification revealed itself important for the enzyme fixing on the PPB film as well as for maintaining its activity upon immobilization [83]. Both CuNiR/PPB/PBV/GCE and c*c*NiR/PPyV/GCE biosensors were characterized by cyclic voltammetry (Table 2) under anaerobic conditions since oxygen interferences were detected. Regarding stability, the c*c*NiR/PPyV/GCE biosensor gave a steady response over four days, neither losing catalytic or redox activity; after this period the signals of the polymerized viologen and of the biocatalyst continuously decreased, but protein denaturation appeared to be the most relevant factor contributing for the response decay [82]. The storage stability of the CuNiR/PPB/PBV/GCE bioelectrode was found to decrease from 93.8% to 85% from day two to day three [83].

(14) $${\text{NO}}_{2}^{-}+8{\text{H}}^{+}+3{\text{H}}_{2}\text{AQS}\to {\text{NH}}_{4}^{+}+2{\text{H}}_{2}\text{O}+3\text{AQS}+6{\text{H}}^{+}$$

(15) $${\text{NO}}_{2}^{-}+8{\text{H}}^{+}+6{\text{V}}^{+.}\to {\text{NH}}_{4}^{+}+2{\text{H}}_{2}\text{O}+6{\text{V}}^{2+}$$

(16) $${\text{NO}}_{2}^{-}+2{\text{H}}^{+}+{\text{PPB}}^{+}\to \text{NO}+{\text{H}}_{2}\text{O}+{\text{PPB}}^{2+}$$

##### 2.2.1.2. Physiologic Mediators

More recently, mediated biosensor constructions based on the interaction of NiRs and their natural electron-transfer partner proteins have been proposed [89–91]. This type of mediation may prove advantageous relatively to biosensors based on artificial mediators, given that it mimics more faithfully the electron transfer processes occurring *in vivo*. Very importantly, the avoidance of synthetic electronic mediators (which are frequently hazardous and general catalysts) turns the method more environmental friendly and improves detection selectivity. As below described, the bioreceptor/mediator couples CuNiR/pseudoazurine from *A. faecalis* and *cd*_1_NiR/cytochrome-*c*_552_ (cyt-*c*_552_) purified from *Marinobacter hydrocarbonoclausticus* (*M. hydrocarbonoclausticus*) were used in such approach. Furthermore, Loujou *et al.* have also shown that the catalytic activity of *cd*_1_NiR from *Pseudomonas aeruginosa* could be monitored by cyclic voltammetry if the ET reaction was coupled to the putative redox partners cytochrome *c*_551_ or azurin, which behaved as fast electrochemical systems (surprisingly, no electrocatalytic activity was detected when artificial electron shuttles were employed) [92].

CuNiR and pseudoazurine (PAz) were associated in a study conducted by Astier *et al.* foreseeing future applications in the construction of biosensor devices [89]. Gold electrodes were used as the transducing element, the surface of which was made more biocompatible through modification with cysteine-containing hexapeptides. In the presence of the CuNiR, pseudoazurine and nitrite, all in solution, both heterogeneous and intermolecular electron transfer occurred, enabling the detection of enzyme turnover. The authors also tested an inorganic mediator, ruthenium hexamine, instead of the pseudoazurine for enzyme mediation, but a more limited linear range of 1–100 μM (compared to 0–1,500 μM) was attained (see Table 2) [89]. Although both mediating and biorecognition molecules were not attached to the transducing electrode, the principle was innovative, launching the concept of coupling ET between enzymes and their physiological redox partners for the development of efficient nitrite biosensors. In the same line of work, in a quite recent report, Tepper has immobilized the same set of proteins on a SAM-modified gold electrode using specific DNA tethers [90]. Both PAz and CuNiR were modified with complementary DNA tags which were allowed to hybridize with the gold anchored SH-DNA strands. This strategy enabled a tight association of the two proteins with the electrode surface and, at the same time, granted appropriate mobility and proximity for electron shuttling and catalytic turnover (Figure 1(c)). Nevertheless, the analytical performance of this proposal was not fully explored; further studies should be done in order to maximize the biosensing potential [90].

Very recently, our group has proposed a nitrite biosensor based on the couple *cd*_1_NiR/cyt-*c*_552_. The co-immobilization of the two proteins was achieved by entrapment within a photopolymerisable polyvinyl alcohol derivative with azide pendant groups (PVA-AWP) matrix. The mixture of all components was deposited on carbon paste screen-printed electrodes (CPSPE) and characterized amperometrically at a working potential of −0.1 V (*vs.* Ag/AgCl) (Figure 1(a)). Cyt-*c*_552_ delivered high-quality mediating properties. In fact, besides favoring ET to *cd*_1_NiR, much reducing unspecific electronic reactions, it also enabled to work at near 0 V (*vs.* Ag/AgCl), therefore avoiding problematic high polarization potentials [91].

#### 2.2.2. Biosensors Based on Direct Electrochemistry

Nitrite biosensors based on the DET between enzymes and electrodes have also been presented (Figure 1(d)) [88,93,94]. The prerequisites for achieving an efficient heterogeneous electron transfer reaction include the correct orientation of the protein molecules on the electrode surface and a sufficiently short distance between the redox active site and the electrode interface. If no tethering system is used to control molecular spatial organization, in addition to enzyme turnover and substrate diffusion, the catalytic current may also reflect the average of individual interfacial ET rates. Very importantly, the operating potential in DET based devices is directly related to the redox potential of the enzyme, usually upgrading selectivity and avoiding interfering reactions. The biosensor’s manufacturing is also simplified since fewer reagents are required.

Thus far, c*c*NiR of *D. desulfuricans* is the only NiR that has been used in DET biosensors, the first results being presented by Scharf and co-workers [88]. In parallel to the mediated approach previously mentioned, using a polyacrilamide gel to entrap the protein on GC electrodes, a direct electrochemical response was additionally observed. The importance of such result was underestimated at the time, and only recently the capacity of c*c*NiR to establish a direct electrochemical communication with carbon electrodes was further exploited in the biosensing domain. In this regard, our group has used pyrolytic graphite electrodes as transducing platforms, which provide a good interface for DET with c*c*NiR. In this way, intense nitrite dependent catalytic currents were displayed. In one study c*c*NiR was incorporated within a sol-gel porous matrix composed of the hydrophobic sol-gel precursor 2-(3,4-epoxycyclohexyl)-ethyltrimethoxy silane (EETMS) [93]. Even though the biosensor was characterized (Table 2) at a quite negative polarization potential (−900 mV *vs.* Ag/AgCl), it was possible to obtain an electrocatalytical response to nitrite at higher potentials; therefore, it offered a chance to work without the interference from oxygen. Nonetheless, when operating at these higher potentials (−500 mV *vs.* Ag/AgCl) the linear range was dramatically shortened (the upper limit decreased from 50 μM to 2 μM). The c*c*NiR/EETMS/PGE sensor response was very fast, reaching maximum currents within 5 s. Tests with river water samples revealed an excellent accuracy when compared to the standard method results. A quite stable response was kept during two weeks on the basis of a daily calibration. Remarkably, the enzyme was active for a much longer period: this biosensor still had some residual electrocatalytical activity after six months [93]. Soon after, a highly sensitive response to nitrite was obtained by depositing a c*c*NiR film atop of single-wall carbon nanotubes (SWCNTs) multilayered on pyrolytic graphite electrodes [94]. In fact, the SWCNTs were able to enhance drastically the sensitivity to nitrite, reaching a record of 2,400 mA·M^−1^·cm^−2^ (Table 2). The SWCNTs electrode modification creates larger surface areas, thus providing more sites for heterogeneous ET and for protein entrapment, as attested by the influence of the number of carbon nanotubes layers on the catalytic response. In terms of stability, better results were obtained when protective coatings such as laponite (an inorganic clay) were deposited over the enzymatic film. In fact, the sensitivity of the laponite/c*c*NiR/SWCNTs/PGE biosensor was fairly reproducible over the period of three months and, as in the previous sol-gel based electrode, enzymatic activity was detected for several months. However, the presence of extra coatings created a diffusion barrier that diminished the biosensor sensitivity in 75% [94].

## 3. Non-Amperometric Based Nitrite Biosensors

### 3.1. Potentiometric Detection

Kiang and collaborators pioneered the report of nitrite biosensors based on nitrite reducing enzymes. In this work, a sirohemic NiR extracted from spinach leaves was incorporated in a co-polymer made up of bovine serum albumine (BSA) and glutaraldehyde and prepared by a freeze-thaw process. Dithionite (S_2_O_4_^2−^) reduced methyl viologen was employed as the enzyme electron donor. The ammonia generated from the nitrite reduction reaction could be detected with a potentiometric electrode (Figure 1(e)), apparently free from other ions interference [95].

### 3.2. Conductimetric Detection

A conductimetric nitrite biosensor was developed by Zhang *et al.* in collaboration with our group, immobilizing the c*c*NiR from *D. desulfuricans* in the presence of BSA, Nafion, methyl viologen, glycerol and glutaraldehyde vapor (as cross-linking agent) [96]. The sensor was prepared by casting the enzyme preparation and a control mixture without c*c*NiR on a pair of gold interdigitated electrodes—the working and the reference electrodes, respectively. The reaction was initiated by the addition of sodium dithionite to a previously purged nitrite containing phosphate buffer solution, in order to reduce the immobilized redox mediator, which in turn further activated c*c*NiR (Figure 1(f)). The reaction generates conductance variation inside the miniaturized cell, which could be measured and correlated with nitrite concentration present in solution. This biosensor displayed a prompt response (10 s) with a linear range and detection limit of 0.2–120 μM and 0.05 μM, respectively. The response was stable for at least one week, gradually dropping after this period. Interference studies showed little or no response (lower than 5%) to common ions present in water. The biosensor was used to determine the nitrite in river water samples with recovery rates between 105–109 % [96].

### 3.3. Optical Detection

Similar works published by Ferreti *et al.* [97] and Rosa *et al.* [98] have suggested the employment of *cd*_1_NiR from *Paracoccus pantotrophus* (*P. pantotrophus*) in optical nitrite biosensors. In both cases, signal transduction was based on the intensity variation of the absorption band assigned to the catalytic *d*_1_-type heme (λ = 460 nm) following nitrite addition. Prior to measurements, the sensors were exposed to sodium dithionite for enzyme activation (Figure 1(g)). In a first report, sol-gel/enzyme matrices were used in two different configurations [97]. Firstly, *cd*_1_NiR was encapsulated in bulk sol-gel monoliths of tetramethyl orthosilicate (TMOS), which were deposited on the optical face of a spectrophotometric cuvette. The sensor had a detection limit of 0.075 μM and a linear range up to 1.250 μM nitrite. Sensitivity was determined to be 7.9 nM^−1^ (Table 2). As an alternative approach, a sol-gel sandwich sensor configuration was investigated where the protein was deposited between two tetraethylorthosilicate (TEOS) sol-gel thin films, enabling to speed up the response time from 15 to 5 minutes [97]. Later on, a second optical *cd*_1_NiR biosensor was proposed using controlled pore glass (CPG) beads of isothiocyanate for enzyme immobilization [98]. Since the matrix caused high levels of radiation scattering, the authors have chosen to obtain signal transduction by visible light diffuse reflectance using a purposely designed apparatus, instead of common spectrophotometric equipments. However, this system showed a poorer analytical performance, with a detection limit of 0.93 μM, a linear range of 0–4 μM and a sensitivity of 18.5 nM^−1^ (Table 2) [98].

## 4. Conclusions and Future Trends

From the comprehensive literature review here presented, it is clear that enzyme based nitrite biosensors represent an important Research and Development field, targeting key applications in the industrial, environmental and biomedical markets. The ability to discriminate the analyte against potentially interfering species due to the integration of selective biocatalysts represents an added value for nitrite screening in food, waters and physiological samples. Owing to the redox nature of the underlying biorecognition event, electrochemical transducers in general, and amperometric/voltammetric ones in particular, are well suited for converting the catalytic reaction into a quantifiable signal. Moreover, the insensitivity of electrochemical methods to medium color and turbidity is a competitive advantage over the much less used optical approaches, whereas potentiometric and conductimetric signal transducers are more prone to interference from ionic species.

Although the construction of these sensing devices is far from trivial, major progresses have been made over the last decade. After the preliminary studies carried out using non-immobilized electron carrier species, fully integrated biosensors based on mediated electrochemistry have become a common configuration. More advanced strategies operating in the unmediated mode via DET [93] and exploiting nanostructured materials as electrodes interfaces were recently proposed [94]. In parallel, stability has been substantially improved through the construction of leak free devices and the use of protecting coats. Very recently, the screen printing technology was successfully employed, opening up the route for miniaturization [91].

Despite the unquestionable usefulness of the reported works, a number of setbacks need to be overcome in order to reach the commercialization phase. For example, the negative reduction potentials generally required to activate nitrite reductases imposes oxygen removal prior to electrochemical assays, therefore hindering *in situ* measurements. On the other hand, the promising trend of coupling electron transfer to the enzymes physiological partners (PAz/CuNiR and cyt-*c*_552_/*cd*_1_NiR) appears to be a consistent solution since the formal reduction potential of these physiological mediators is rather low, benefiting the analysis selectivity either from oxygen interferences or non specific catalysis [89–91].

Enzyme availability may also constitute an economic obstacle to the cost-effective mass production of nitrite biosensors. The construction of alternative microbial biosensors, as already proposed by Nielsen *et al.* [99] and Reshetilov *et al.* [100], could save on time and costs of enzyme purification; but the issue of selectivity gets worst, alongside with practical difficulties associated to the implementation of whole cells biosensors [2]. In order to address the problem of protein production, new working lines on heterologous overexpression of NiRs should be encouraged.

As proved by the works of Wu *et al.* [83] and Sasaki *et al.* [84], protein engineering could also be very useful to tailor the enzyme’s properties (catalytic activity and selectivity) and to both facilitate electron transfer and protein immobilization.

At the moment, there is insufficient knowledge on the catalytic properties of NiRs, no matter the type. Focused research on fundamental understanding of enzyme kinetics would lead to a qualitatively better control and prediction of NiRs’ behavior. To conclude, the future of R&D on nitrite biosensing devices should be settled on stronger collaborative approaches, bringing together expertise in biochemistry, electrochemistry, nanotechnology, materials science, electronics and biotechnology. Such interdisciplinary projects will certainly boost the development of nitrite biosensors, leading to the implementation of a commercial device.

## Figures and Tables

**Table 1.** Description and analytical parameters of nitrite biosensors based on non-specific proteins (N.D. – not determined).

**Protein**	**Source**	**Sensor Preparation**	**Transducer**	**Linear Range**	**Detection Limit**	**Sensitivity**	**Reference**
Mb	horse heart	GC/casting of hexagonal mesoporous silica + PVA + protein	Amperometric	8–216 μM	0.8 μM	0.158 μAμM^−1^	[34]
Mb	horse heart	graphite/nanoporous ZnO electrodeposition/protein dip coating	Amperometric	10–180 μM	4 μM	N.D.	[35]
Mb	horse heart	GC/casting of zirconium phosphate nanosheets films + protein	Amperometric	3–800 mM	700 μM	N.D.	[36]
Mb	equine skeletal muscle	GC/casting of clay-chitosan-gold nanoparticle nanohybrid/casting of protein between matrix layers	Amperometric	2.49–63.3 mM	500 μM	N.D.	[37]
Mb	horse heart	GC/casting of silica-coated gold nanorods + room temperature ionic liquid + silica sol-gel composite film + protein	Amperometric	0.04–5.0 mM	20 μM	N.D.	[38]
Mb	horse heart	carbon paste + ionic liquid/casting of multi-walled carbon nanotubes + Nafion/casting of protein between matrix layers	Voltammetric	0.2–11.0 mM	N.D.	26.9 μA mM^−1^	[39]
Mb	horse heart	pyrolytic graphite/ dip coating of zeolite particles/dip coating of protein between matrix layers.	Voltammetric	50–2,400 μM	5 μM	31.1 μA μM^−1^cm^−2^	[40]
Hb	bovine			50–2,200 μM	5 μM	23.5 μA μM^−1^ cm^−2^	
Hb	--------------	carbon paste + gold nanoparticles + protein	Voltammetric	1–9.7 μM	0.06 μM	0.071 μA μM^−1^	[41]
Hb	bovine blood	GC/casting of hexagonal mesoporous silica + PVA + protein	Amperometric	0.2–3.8 μM	0.61 μM	1.79 μA μM^−1^	[42]
Hb	bovine blood	GC/casting of colloidal silver nanoparticles + protein/vapor deposition of titania sol-gel	Amperometric	0.2–6.0 mM	34 μM	−5.84 μA mM^−1^ cm^−2^	[43]
Hb	bovine blood	GC/casting of CdS nanospheres + Nafion + protein	Amperometric	0.3–182 μM	0.08 μM	N.D.	[44]
Hb	bovine blood	GC/protein electrospin followed by cross linking with glutaraldehyde	Amperometric	N.D.–4.5mM	0.47 μM	N.D.	[45]
HRP	horseradish	GC/ casting of gemini surfactant C_12_-C_12_-C_12_ + PVA + protein	Voltammetric	0.03–12 mM	N.D.	−0.0302 μA mM^−1^	[51]
Mb	horse heart	graphite/casting of silk fibroin + protein	Voltammetric	235–13,600 μM	N.D.	9.95 μA mM^−1^	[46]
Hb	------------			233–14,700 μM	N.D.	8.70 μA mM^−1^	
HRP	horseradish			1.85–13.8 mM	N.D.	8.21 μA mM^−1^	
Cat	bovine liver			2.52–13.8 mM	N.D.	8.63 μA mM^−1^	

**Table 2.** Description and analytical parameters of nitrite reductase based biosensors

**Enzyme**	**Source**	**Sensor preparation**	**Transducing mode**	**Electron transfer**	**Linear Range**	**Detection Limit**	**Sensitivity**	**Reference**
Sirohemic NiR	Spinach leafs	enzyme + BSA + glutaraldehyde	Potentiometric	N.A.	0.1–50 mM	N.D.	N.D.	[95]
c*c*NiR	*D. desulfuricans*	GC/casting of enzyme + polyacrylamide (mediator in solution)	Voltammetric	MET (methyl viologen)	up to 200 μM	N.D.	N.D.	[88]
c*c*NiR	*D. desulfuricans*	GC/casting of enzyme + polyacrilamide		DET	up to 200 μM	N.D.	N.D.	
c*c*NiR	*D. desulfuricans*	GC/dispersion of poly(pyrrole-viologen) + enzyme mixture followed by electropolymerization	Voltammetric	MET (poly(pyrrole-viologen))	5.4–43.4 μM	5.4 μM	1,721 mA M^−1^cm^−2^	[82]
c*c*NiR	*D. desulfuricans*	GC/casting of Nafion + enzyme/ incorporation of mediator	Voltammetric	MET (methyl viologen)	75–800 μM	60 μM	445 mA M^−1^cm^−2^	[30]
c*c*NiR	*D. desulfuricans*	GC/casting of [ZnCr-AQS] LHD + enzyme/glutaraldehyde vapor cross-linking	Amperometric	MET (AQS)	0.015–2.350 μM	4 nM	1,824 mA M^−1^cm^−2^	[85]
c*c*NiR	*D. desulfuricans*	gold/casting of Nafion + enzyme + mediator + glycerol + BSA/ glutaraldehyde vapor cross-linking	Conductimetric	MET (methyl viologen)	0.2–120 μM	0.05 μM	0.194 μS/μM	[96]
c*c*NiR	*D. desulfuricans*	pyrolytic graphite/casting of EETMS sol/ casting of enzyme	Amperometric	DET	0.25–50 μM	120 nM	430 mA M^−1^cm^−2^	[93]
c*c*NiR	*D. desulfuricans*	graphite/casting of SWCNTs dispersion/casting of enzyme	Voltammetric	DET	up to 150 μM	N.D.	2,400 mA M^−1^cm^−2^	[94]
c*c*NiR	*S. deleyianum*	graphite and mediator composite/casting of enzyme + poly(carbamoyl sulfonate) hydrogel membrane	Amperometric	MET (phenosafranin)	up to 250 μM	1 μM	446.5 mA M^−1^cm^−2^	[80]
*cd*_1_NiR	*P. denitrificans*	graphite/enzyme entrapment through dialysis membrane (mediator in solution)	Amperometric	MET (1-methoxy PMS)	4.35–65.2 μM*	N.D.	N.D.	[81]
*cd*_1_NiR	*P. denitrificans*	graphite/enzyme entrapment with dialysis membrane (mediator in solution)	Amperometric	MET (1-methoxy PMS)	up to 750 μM	10 μM	33 mA M^−1^cm^−2^	[80]
*cd*_1_NiR	*P. pantotrophus*	enzyme incorporated in bulk sol-gel monoliths of TEOS	Optical	N.A.	0.075–1.250 μM	0.075 μM	N.D.	[97]
*cd*_1_NiR	*P. pantotrophus*	enzyme in controlled pore glass beads of isothiocyanate	Optical	N.A.	0–4 mM	0.93 μM	19.5 nM^−1^	[98]
*cd*_1_NiR	*M. hydrocarbonoclausticus*	graphite/casting of polyvinyl alcohol + enzyme + mediator followed by photopolymerization	Amperometric	MET (cyt-*c*_552_)	10–200 μM	7 μM	2.49 A cm^2^ μM^−1^	[91]
CuNiR	*R. sphaeroides*	GC/electropolimerization of PPB/ casting of enzyme + PBV	Voltammetric	MET (PPB)	up to 50 μM	1 μM	789 mA M^−1^cm^−2^*	[83]
CuNiR	*R. sphaeroides*	GC/casting of poly(vinyl alcohol) + mediator + enzyme/ casting of poly(allylamine hydrochloride)/casting of hydrophilic polyurethane	Amperometric	MET (methyl viologen)	1.5–260 μM	1.5 μM	170 mA M^−1^cm^−2^*	[79]
CuNiR	*A. faecalis*	gold/enzyme entrapped with dialysis membrane (mediator in solution)	Amperometric	MET (1-methoxy PMS)	0–22 μM*	0.22 μM*	N.D.	[84]
CuNiR	*A. faecalis*	gold/dip-coating in (cysteine) thiolated hexapeptide (enzyme and mediator in solution)	Voltammetric	MET (pseudoazurine)	200–1,500 μM	N.D.	N.D.	[89]
		gold/dip-coating in (cysteine) thiolated hexapeptide (enzyme and mediator in solution)		MET (ruthenium hexamine)	1–100 μM	N.D.	N.D.	

(N.A.—not applicable; N.D.—not determined; MET—mediated electron transfer; DET—direct electron transfer;

* —original values were converted to the same final unit).
